# Evaluation of Multiple Influences on the Unconfined Compressive Strength of Fibre-Reinforced Backfill Using a GWO–LGBM Model

**DOI:** 10.3390/ma19010200

**Published:** 2026-01-05

**Authors:** Xin Chen, Yunmin Wang, Shengjun Miao, Shian Zhang, Zhi Yu, Linfeng Du

**Affiliations:** 1 Sinosteel Maanshan General Institute of Mining Research Co., Ltd., Ma’anshan 243000, China; chenxin_ck@csu.edu.cn (X.C.);; 2 School of Resources and Safety Engineering, Central South University, Changsha 410083, China; 3 School of Resources and Safety Engineering, University of Science and Technology Beijing, Beijing 100083, China; 4 Zijin School of Geology and Mining, Fuzhou University, Fuzhou 350116, China

**Keywords:** fibre-reinforced cemented paste backfill, unconfined compressive strength, Light Gradient Boosting Machine, Grey Wolf Optimizer, SHapley Additive exPlanations

## Abstract

**Highlights:**

**What are the main findings?**

- A GWO–LGBM model is proposed for the high-accuracy prediction of UCS in fibre-reinforced CPB.
- Multi-factor analysis is applied to evaluate the coupled effects of mixture proportions, the tailings’ physical/chemical characteristics, and fibre properties on UCS.

**What are the implications of the main findings?**

- Cement content and curvature coefficient are identified as the primary factors affecting the UCS of CPB.
- Fibres are found to enhance structural bonding, whereas the chemical composition of tailings plays an inert role.

**Abstract:**

Fibres can markedly enhance the uniaxial compressive strength (UCS) of cemented paste backfill (CPB). However, previous studies have mainly verified the effectiveness of polypropylene and straw fibres in improving the UCS of CPB experimentally, while systematic multi-factor evaluation remains limited. In this study, laboratory experiments were conducted on polypropylene- and straw fibre-reinforced CPB to construct a reliable dataset. The factors influencing the intensity of uniaxial compressive strength were divided into four aspects (mixture proportions, physical properties of the cement–tailings mixture, chemical characteristics of tailings, and fibre properties), and four intelligent models were developed for effectiveness analysis and UCS prediction. SHapley Additive exPlanations (SHAP) were employed to quantify the contributions of individual features, and the findings were experimentally validated. The GWO–LGBM model outperformed the SVR, ANN, and LGBM models, achieving *R*^2^ = 0.907, *RMSE* = 0.78, *MAE* = 0.515, and *MAPE* = 0.157 for the training set, and *R*^2^ = 0.949, *RMSE* = 0.627, *MAE* = 0.38, and *MAPE* = 0.115 for the testing set, respectively. Feature analysis reveals that mixture proportions contribute the most to UCS, followed by the tailings’ physical properties, the fibre properties, and the tailings’ chemical characteristics. This study found that cement content and tailings gradation control CPB structural compactness and fibres enhance bonding between hydration products and tailings aggregates, while the chemical composition of the tailings plays an inert role, functioning mainly as an aggregate.

## 1. Introduction

Cemented paste backfill (CPB) has been widely adopted around the world owing to its notable advantages affecting mining safety, resource recovery, and environmental protection [1,2,3]. These advantages are fundamentally based on the mechanical performance of CPB, among which uniaxial compressive strength (UCS) is regarded as the key indicator for quality assessment [4,5,6]. To further enhance the UCS of CPB, fibres have been increasingly introduced into CPB mixtures and have demonstrated considerable reinforcement potential [7,8,9]. However, previous studies have largely focused on verifying the macroscopic performance improvement of fibre-reinforced CPB, while its mechanical behaviour remains insufficiently understood. Therefore, a comprehensive understanding of the factors affecting the UCS of fibre-reinforced CPB is important for its design and application.

At present, the literature on the strength behaviour of fibre-reinforced CPB remains predominantly based on laboratory experiments. Previous studies have primarily investigated the effects of cement, tailings, and fibre contents, and confirmed these parameters as key factors governing the UCS of fibre-reinforced CPB [10,11,12]. However, besides these conventional factors, other factors, such as particle size distribution [13,14], sulphide content [15,16,17], and major oxide composition [18], also exert a significant influence on the UCS of CPB. The effects of these properties on UCS are often nonlinear and non-monotonic, meaning that they may either promote or inhibit strength development depending on their specific ranges. Nevertheless, such physical and chemical properties of tailings have been rarely addressed in experimental studies. On the one hand, laboratory experiments waste time and energy, making it impossible to cover a wide range of influencing factors comprehensively. On the other hand, conventional analytical approaches are limited in their ability to extract meaningful insights from relatively small datasets and to capture the interactions among multiple variables. These limitations, to some extent, constrain the depth and generality of conclusions derived from purely experimental investigations.

To better understand this multifaceted system, researchers have developed many approaches. Various empirical models have been proposed to describe the parameters which effect UCS, e.g., the effects of mass concentration [19], cement–tailings ratio [20], and fibre properties [21]. However, these empirical models generally focus on a single variable and are unable to account for interactions among multiple parameters, limiting their applicability. With the development of machine learning (ML), new opportunities have emerged for analysing nonlinear and strongly coupled systems [22,23,24,25]. For instance, Qi et al. [26] achieved high-accuracy UCS predictions for CPB using a PSO–ANN (Particle Swarm Optimisation–Artificial Neural Network) algorithm and explored the combined effects of tailings type, cement–tailings ratio, solids content, and curing time. Yu et al. [27] applied an SSA–ELM (Sparrow Search Algorithm–Extreme Learning Machine) model and identified fibre content and fibre length as major contributors to the UCS of fibre-reinforced CPB. Arachchilage et al. [28] tested four ML methods to predict the UCS of alkali-activated slag-based CPB and reported good agreement between predictions and experiments, highlighting curing time and water–binder ratio as dominant factors. Xi et al. [29] successfully employed the LGBM (Light Gradient Boosting Machine) model to predict UCS of recycled aggregate concrete. Although ML techniques have shown considerable promise in analysing the mechanical behaviour of CPB, the input variables used in most studies remain largely limited to conventional experimental parameters, with the physical and chemical properties of tailings rarely considered. In addition, LGBM models optimised using Grey Wolf Optimisation (GWO) have not yet been reported for CPB strength prediction.

To address these limitations, it is necessary to evaluate the UCS of fibre-reinforced CPB through experiments and a new high-accuracy GWO–LGBM model. A series of pro-portioning experiments considering conventional backfill ratio parameters and fibre parameters were conducted to verify the effectiveness of fibre reinforcement and obtain reliable datasets. The dataset used herein is categorised into four groups: mixture proportions; physical properties of the cement–tailings mixture; chemical characteristics of tailings; and fibre properties. This framework enables a comprehensive analysis of the contributions of different parameters to the UCS of CPB, providing valuable theoretical insights into the mechanisms governing its mechanical behaviour.

## 2. Materials and Mechanical Tests

### 2.1. Materials

The materials required for backfilling comprise the following: (1) Aggregates: Sourced from a lead–zinc mine in southern China. Two types were used: classified tailings and rod-mill tailings. The rod-mill tailings were incorporated to improve the particle size distribution of the classified tailings. (2) Binder: Ordinary Portland Cement (PO.42.5). (3) Fibres: Polypropylene fibre and straw fibre. (4) Water: Tap water.

**Figure 1:**
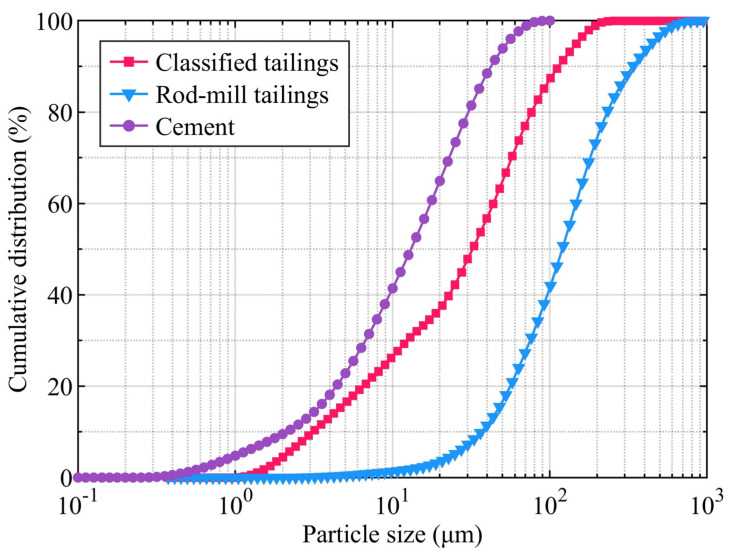
Particle size distribution of cement and tailings.

The particle density of the two types of tailings and the cement was first determined using the Le Chatelier flask method. Subsequently, the particle size distribution of the tailings and cement was analysed with a laser particle-size analyser (MLS13320, Beckman, Brea, CA, USA); the results are presented in Figure 1.

The results indicate that the cement had a particle density of 3.11 t/m^3^ and a specific surface area of 1.25 m^2^/g. The cement particles were relatively fine, with over 64.93% of particles being smaller than 20 µm. The classified tailings exhibited a particle density of 3.18 t/m^3^, with a median particle size (*D*_50_) of 35.42 µm, an average particle size (*D*ₐᵥ) of 50.59 µm, and characteristic sizes of *D*_10_ = 3.206 µm, *D*_30_ = 12.99 µm, and *D*_60_ = 47.94 µm. For the rod-mill tailings, these values were 2.8 t/m^3^, 131.8 µm, 173.0 µm, 39.78 µm, *D*_30_ = 76.43 µm, and 146.8 µm, respectively. To quantitatively evaluate the particle size distribution of the cement–tailings mixture, the uniformity coefficient (UC) and the curvature coefficient (CUC) were employed as follows:

(1) $$\mathrm{UC}=\frac{D_{60}}{D_{10}}$$

(2) $$\mathrm{CUC}=\frac{{D_{30}}^{2}}{D_{10}\times D_{60}}$$

The chemical composition of the two types of tailings was measured using X-ray fluorescence (XRF) spectroscopy (ZSX Primus III, Rigaku Corporation, Tokyo, Japan), with the results given in Table 1. According to the XRF measurements, Fe, Ca and Al are the main metallic elements, and heavy metals occur only in trace amounts. Overall, the elemental composition of the two tailings types is broadly similar.

The main non-metallic oxide is SiO_2_, with contents of 16.80% in the classified tailings and 24.60% in the rod-mill tailings. The primary metallic oxides are Al_2_O_3_, Fe_2_O_3_, MgO, CaO, and K_2_O, while other oxides appear in minor quantities. The relatively high proportions of SiO_2_ and Al_2_O_3_ indicate that it is a suitably inert material, and the significant presence of CaO and a certain amount of MgO is conducive to the development of the binding properties of the cemented backfill. By contrast, the rod-mill tailings contain more sulphur, which is unfavourable for strength gain and can impair backfill quality. To quantify the chemical characteristics of the tailings, four indices were adopted: sulphur content S, alkaline-to-acidic oxide ratio M ((CaO + MgO)/(SiO_2_ ± TiO_2_)), oxide mass coefficient K ((CaO + MgO + Al_2_O_3_)/(SiO_2_ + MnO + TiO_2_)), and alkali coefficient B ((CaO + MgO + Al_2_O_3_)/SiO_2_).

Monofilament polypropylene (C_3_H_6_) and straw fibres were incorporated into the CPB to enhance its strength. The polypropylene fibre is primarily composed of carbon and has a single filament diameter of 0.19 mm, a linear density of 0.91 g/cm^3^, an elastic modulus of 3.5 GPa, and a fracture strength of 350 MPa. Microscopic observations indicate that the fibre surface is smooth and uniform, with few adhered particles. It offers the advantages of excellent toughness, tensile and crack resistance, low cost, and good self-dispersibility, allowing it to be easily and uniformly distributed within the slurry. Straw fibre, commonly treated as an agricultural waste product, contains a significant amount of cellulosic material and also exhibits certain mechanical properties and chemical stability, with an elastic modulus and fracture strength of 20 GPa and 50 MPa, respectively. Its main elemental components are carbon and oxygen, with small amounts of silicon and potassium. Microscopic observations reveal that the surface of straw fibre is relatively rough and covered with irregular protrusions of varying sizes, which are beneficial for increasing interfacial friction between the fibre and the surrounding matrix. For this experiment, four different lengths of polypropylene fibre—3, 6, 9, and 12 mm—were used as one set of variables. Similarly, straw fibre was categorised into three different length classifications—0.8, 2, and 4 cm—for study.

### 2.2. Mechanical Test

**Figure 2:**
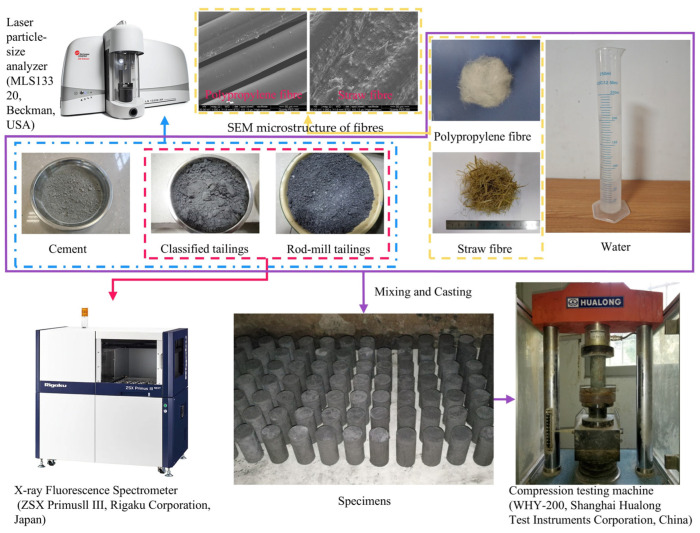
Experimental flowchart.

Considering the different strength requirements of CPB for stope and pillar mining conditions, an orthogonal experimental design was adopted, and the experimental procedure is illustrated in Figure 2. Two separate experimental schemes were established for the polypropylene fibre group and the straw fibre group, using *L*_16_ (4^5^) and *L*_18_ (3^5^) orthogonal arrays, respectively. Five factors were considered: cement-to-tailings mass ratio (A), solids concentration (B), fibre content (C), fibre length (D), and the proportion of rod-mill tailings (E). For the polypropylene fibre group, the factor levels were set as follows: A1–A4: 1:3, 1:4, 1:5, and 1:6 for stope backfilling, and 1:8, 1:10, 1:12, and 1:14 for pillar backfilling; B1–B4: 73%, 75%, 77%, and 79%; C1–C4: 0%, 0.05%, 0.10%, and 0.15%; D1–D4: 0, 0.5, 1.0, 1.5, and 2.0 kg/m^3^; E1–E4: 3, 6, 9, and 12 mm. For the straw fibre group, the factor levels were set as follows: A1–A3: 1:4, 1:5, and 1:6 for stope backfilling, and 1:8, 1:10, and 1:12 for pillar backfilling; B1–B3: 73%, 75%, and 77%; C1–C3: 0%, 0.05%, and 0.10%; D1–D3: 0.5, 1.0, and 1.5 kg/m^3^; E1–E3: 0.8, 2.0, and 4.0 cm.

The constituent materials—including the two types of tailings, fibres, cement, and water—were mixed in their designated proportions and agitated thoroughly for 10 min to produce a homogeneous fresh slurry. The slurry was subsequently cast into cylindrical plastic moulds with dimensions of 50 mm in diameter and 100 mm in height. After moulding, all samples were placed in a curing box maintained at roughly 20 °C and about 95% relative humidity, following the procedures specified in GB/T 17671-2021 [31]. UCS tests were performed on the specimens after curing periods of 3, 7, and 28 days, in accordance with ASTM D7012 [32]. The tests were conducted using a compression testing machine (WHY-200, Hualong Test Instrument Corporation, Shanghai, China). During loading, displacement was applied at 0.2 mm/min, and both stress and deformation were logged every second until failure. A total of 487 UCS data points were collected, forming the dataset for subsequent intelligent prediction modelling.

**Figure 3:**
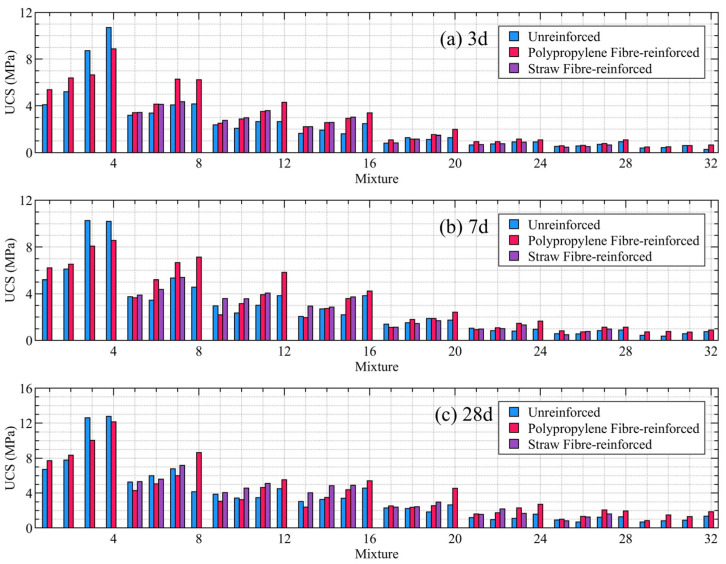
Test result of UCS.

The test results, as illustrated in Figure 3, demonstrate that the incorporation of either polypropylene or straw fibres significantly enhances the compressive strength of the CPB. Over 80% of the fibre-reinforced specimens exhibited a higher UCS compared to the conventional, non-fibrous specimens. This overall improvement in strength indicates that the flexible reinforcement mechanism provided by the fibrous materials effectively enhances the integral stability of the CPB.

### 2.3. Data Description

Previous research on the cementation mechanisms of backfill has predominantly focused on the influence of mixture proportions on mechanical performance [33]. However, as cement and tailings constitute the primary components of CPB, it is essential to consider the role of their physical and chemical properties. Furthermore, analysing the effect of fibre additives is indispensable for a comprehensive understanding of fibre-reinforced CPB performance.

**Figure 4:**
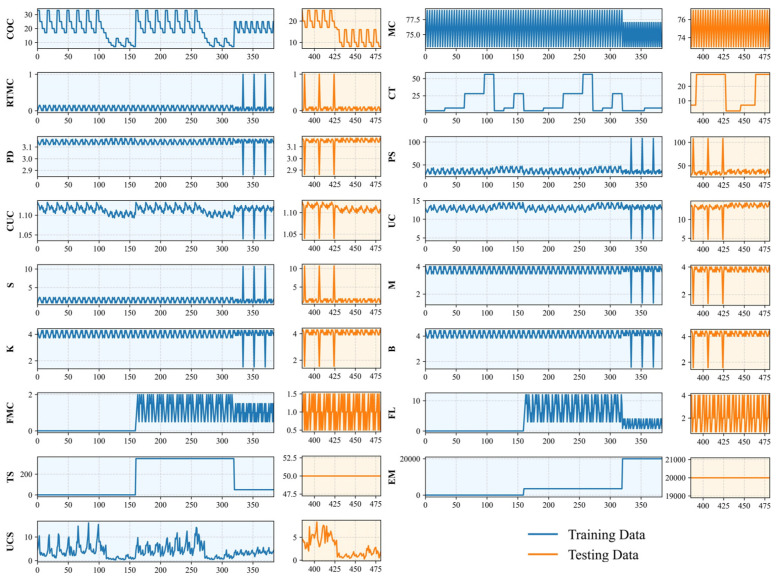
Database of fibre-reinforced CPB.

**Figure 5:**
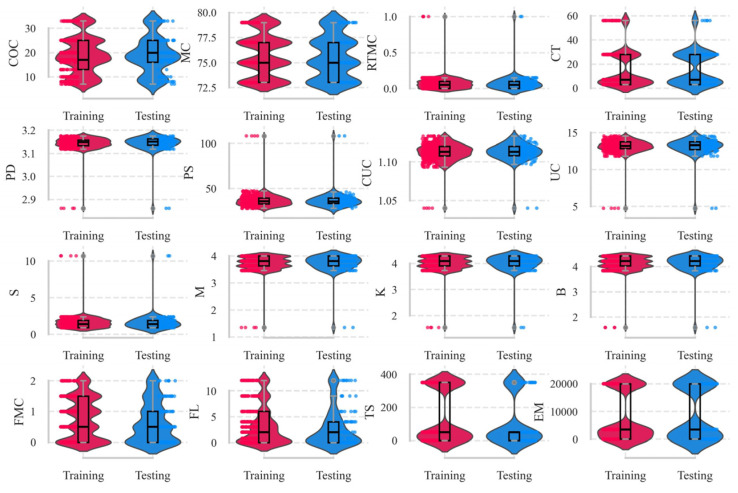
Violin plots of fibre-reinforced CPB data.

Therefore, to systematically account for the influences of mix proportions and the physical and chemical properties of the materials during CPB preparation—with particular emphasis on the role of fibrous additives—the relevant parameters were categorised into four groups: (1) Mixture Proportions: cement content (COC), mass concentration (MC), rod-mill tailings mass concentration (RTMC), and curing time (CT); (2) Physical Properties of the Cement–Tailings Mixture: particle density (PD), particle size D50 (PS), curvature coefficient (CUC), and uniformity coefficient (UC); (3) Chemical Characteristics of Tailings: sulphur content (S), alkaline-to-acidic oxide ratio (M), oxide mass coefficient (K), and alkali coefficient (B); (4) Fibre Properties: fibre mass concentration (FMC), fibre length (FL), tensile strength (TS), and elastic modulus (EM). In this study, the UCS dataset obtained from laboratory experiments was utilised and machine-learning techniques were employed to analyse the influence of these factors and to predict UCS of CPB. The database was divided into training and testing sets at a ratio of 80% to 20%, as shown in Figure 4. The distributions of each variable in the training and testing sets were compared using violin plots (Figure 5). The results indicate that both datasets exhibit similar distribution characteristics, suggesting that the data partition is reasonable and suitable for model training and validation.

## 3. Methods

To establish a high-performance predictive model, three representative machine-learning algorithms—Support Vector Regression (SVR), Artificial Neural Network (ANN), and Light Gradient Boosting Machine (LGBM)—were employed. These were chosen because they embody fundamentally different modelling principles: SVR builds on statistical learning theory, ANN relies on multilayer feed-forward neural architecture, and LGBM uses an efficient gradient-boosting framework [34,35,36]. Their contrasting mechanisms allow a comprehensive evaluation of model capability in capturing the complex nonlinear relationships governing the UCS of fibre-reinforced CPB.

After evaluating the three above models, the one with the highest forecasting capability was chosen for further enhancement through hyperparameter optimisation. Among various metaheuristic methods, Grey Wolf Optimizer (GWO) has proven to be highly effective and widely applied across diverse fields [37]; therefore, it was adopted in this study to enable the base model to achieve superior predictive performance for UCS of fibre-reinforced CPB.

### 3.1. Machine-Learning Methods

#### 3.1.1. Support Vector Regression (SVR)

Support Vector Machine (SVM) was initially proposed by Cortes and Vapnik [38] for classification, and was subsequently extended to regression, forming the SVR algorithm. This method is grounded in statistical learning theory. SVR maps the input data into a higher-dimensional domain and determines a separating function that captures nonlinear patterns using kernel techniques. Among these, the Gaussian Radial Basis Function (RBF) kernel is widely adopted due to its excellent generalisation performance. SVR is particularly suitable for predicting the UCS of CPB characterised by limited sample sizes and pronounced nonlinearity [39,40].

#### 3.1.2. Artificial Neural Networks (ANN)

Originating from the work of McCulloch and Pitts in 1943 [41], ANN emulates neuronal connections in biological nervous systems to process information and learn complex patterns. It represents nonlinear input–output relationships through interconnected neurons arranged in input, hidden, and output layers. During the training process, the model continuously optimises network performance by adjusting connection weights and biases to approximate the actual target values. The Backpropagation (BP) algorithm is one of the most commonly used training methods for ANNs. The input variables and the UCS of CPB exhibit highly nonlinear input–output relationships. Owing to their capacity to model complex, nonlinear systems, ANNs are particularly well suited for predicting the UCS of CPB [23,42,43,44].

#### 3.1.3. Light Gradient Boosting Machine (LGBM)

LGBM, developed by Microsoft Research [45], builds upon the Gradient Boosting Decision Tree framework by incorporating several computational improvements. In CPB strength prediction, the input variables span mixture proportions, tailings physical/chemical properties, and fibre characteristics, resulting in high-dimensional and heterogeneous feature spaces. LGBM addresses these challenges through histogram-based feature discretisation and a leaf-wise tree growth strategy, allowing it to efficiently capture complex nonlinear interactions among CPB components while maintaining low computational cost. Compared with traditional boosting algorithms, LGBM demonstrates superior learning efficiency and generalisation performance when modelling multivariate material behaviour, making it particularly suitable for UCS prediction in fibre-reinforced CPB systems [29,36].

#### 3.1.4. Grey Wolf Optimizer (GWO)

GWO, proposed by Mirjalili et al. [46], mimics the hierarchical structure and collaborative predatory behaviour observed in grey wolf groups. In the context of CPB strength prediction, the nonlinear coupling among mixture proportions, tailings properties, and fibre parameters leads to a complex and non-convex hyperparameter search space for machine-learning models. In GWO, candidate solutions are ranked as *α*, *β*, and *δ* wolves, which guide the search, while the remaining ω wolves follow their lead. The optimisation is achieved through three key behaviours—encircling, hunting, and searching for prey—mathematically implemented by iteratively updating the positions of *α*, *β*, and *δ*, and directing *ω* wolves towards them. This mechanism allows GWO to efficiently balance global exploration and local exploitation, enabling effective tuning of model hyperparameters in complex nonlinear optimisation tasks.

#### 3.1.5. GWO–LGBM

**Figure 6:**
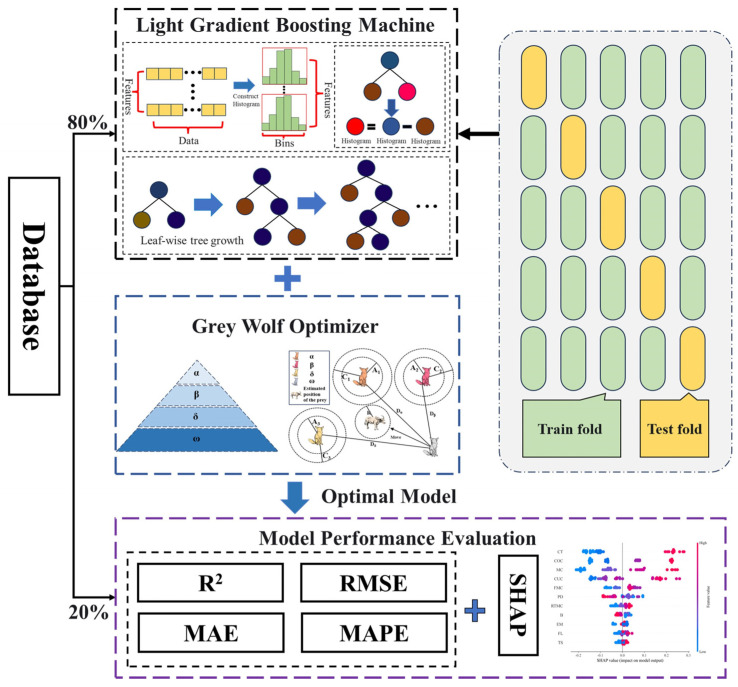
Flowchart of the hybrid prediction model.

The computational procedure of the GWO–LGBM model can be divided into the following six steps (as illustrated in Figure 6):

1. Database Establishment. Construct a database for the UCS of fibre-reinforced CPB, containing 11 input variables and 1 output variable (UCS).
2. Data Pre-processing. Normalise the input and output variables by linearly scaling them to the range [−1, 1] to mitigate scale differences and enhance model training stability.
3. Data Division. The dataset is randomly partitioned, allocating 80% for training and the remaining 20% for subsequent testing. The former subset supports parameter fitting, whereas the latter provides independent evidence of predictive performance.
4. Parameter Optimisation. Employ GWO to globally optimise the key hyperparameters of the LGBM model (such as learning rate, number of trees, and feature sampling rate) to obtain the optimal parameter combination.
5. Model Training. Using the optimised parameter configuration, build the GWO–LGBM model with the training dataset to establish the mapping relationship between nonlinear features and UCS.
6. Performance Evaluation. Validate the model using the testing dataset and assess prediction accuracy and generalisation capability through metrics such as the coefficient of determination (R^2^), root mean square error (RMSE), mean absolute error (MAE), and mean absolute percentage error (MAPE).

### 3.2. Performance Metrics

To quantify the predictive quality, and with reference to previous research findings [47], four metrics were employed to evaluate model performance in this study: the coefficient of determination (*R*^2^), root mean square error (*RMSE*), mean absolute error (*MAE*), and mean absolute percentage error (*MAPE*). Their computational formulas are presented below:

(3) $$R^{2}=1-\frac{\sum_{i=1}^{n}{\left(y_{i}-{\hat{y}}_{i}\right)}^{2}}{\sum_{i=1}^{n}{\left(y_{i}-{\bar{y}}_{i}\right)}^{2}}$$

(4) $$RMSE=\sqrt{\frac{1}{n}\sum_{i=1}^{n}{\left(y_{i}-{\hat{y}}_{i}\right)}^{2}}$$

(5) $$MAE=\frac{1}{n}\sum_{i=1}^{n}\left|y_{i}-{\hat{y}}_{i}\right|$$

(6) $$MAPE=\frac{100\%}{n}\sum_{i=1}^{n}\left|\frac{y_{i}-{\hat{y}}_{i}}{y_{i}}\right|$$

where *n* represents the number of samples used; *ŷᵢ* and *yᵢ* denote the predicted and true values of the target parameter, respectively; and *ȳ* signifies the mean of the true values. For these predictive models, the optimal performance is achieved when *R*^2^ is 1, and *RMSE*, *MAE*, and *MAPE* are 0.

## 4. Result and Analysis

### 4.1. Result of SVR

**Figure 7:**
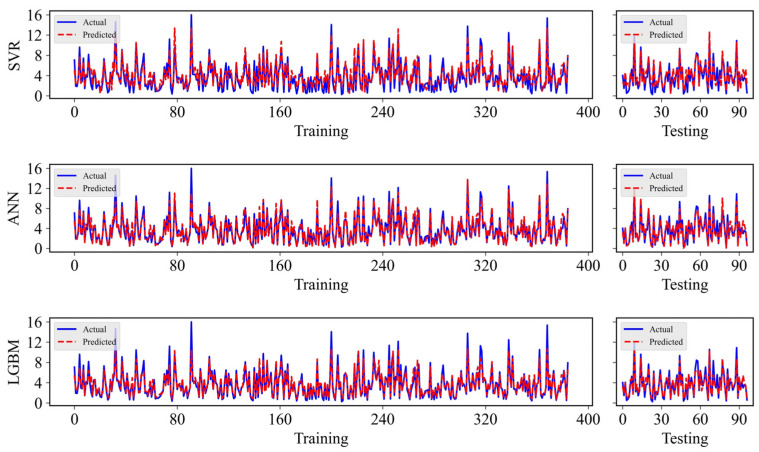
Predicted performance of the SVR, ANN, and LGBM models.

The key hyperparameters of the SVR model include the penalty factor *C*, the insensitive loss coefficient *ε*, and the parameter *γ* in the RBF kernel. Specifically, *C* balances model complexity and training error, *ε* sets the no-penalty error margin, and *γ* controls the influence of each sample. In this study, a grid search strategy was adopted to optimise the SVR hyperparameters, with *C* searched in the range of 0.1–100 and *ε* in the range of 0.01–1, while the RBF kernel was employed. The optimal configuration was determined as: kernel = ‘rbf’, penalty parameter *C* = 10, insensitive loss parameter *ε* = 0.2, and kernel coefficient *γ* = ‘scale’. Based on this parameter setting, the established SVR model achieved satisfactory predictive performance. In terms of conventional evaluation metrics, the established SVR model achieved a prediction performance of *R*^2^ = 0.82, *RMSE* = 1.083, *MAE* = 0.873, and *MAPE* = 0.529 on the training dataset, while the testing dataset yielded *R*^2^ = 0.845, *RMSE* = 1.097, *MAE* = 0.893, and *MAPE* = 0.497 (see Figure 7).

### 4.2. Result of ANN

The ANNs learn the nonlinear relationships between inputs and outputs by mimicking the behaviour of biological neural systems. In this study, a multilayer perceptron (MLP) architecture was employed to construct the ANN model for predicting the UCS of CPB. A systematic investigation was first carried out on the number of hidden layers and the number of neurons (NON) in each layer. The tested architectures include hidden_layer_sizes: (16), (32), (64), (32, 16), (64, 32), and (64, 32, 16). The optimal network architecture was determined as comprising two hidden layers with 64 and 32 neurons. The Rectified Linear Unit (ReLU) was selected as the activation function, as it effectively alleviates the vanishing gradient problem and enhances the network’s ability to capture nonlinear patterns. The Adam optimisation algorithm was adopted for updating the network weights. By combining adaptive learning rates with momentum terms, Adam achieves fast convergence while maintaining stable training behaviour. With the optimised hyperparameters, the ANN model achieved a satisfactory predictive performance. In terms of conventional evaluation metrics, the ANN model yielded *R*^2^ = 0.888, *RMSE* = 0.854, *MAE* = 0.648, and *MAPE* = 0.229 on the training set, whereas the model attained *R*^2^ = 0.898, *RMSE* = 0.89, *MAE* = 0.589, and *MAPE* = 0.2 on the testing set (see Figure 7).

### 4.3. Result of LGBM

For the LGBM model, the hyperparameters were optimised using a grid search strategy. The explored parameter ranges include learning_rate from 0.01 to 0.15, n_estimators from 50 to 200, and colsample_bytree from 0.5 to 1.0. The optimal hyperparameter combination was determined to be learning_rate = 0.1, n_estimators = 120, and colsample_bytree = 0.9. The LGBM model exhibited excellent predictive accuracy. In terms of conventional performance metrics, the LGBM model achieved *R*^2^ = 0.89, *RMSE* = 0.849, *MAE* = 0.606, and *MAPE* = 0.258 on the training set, and *R*^2^ = 0.894, *RMSE* = 0.91, *MAE* = 0.582, and *MAPE* = 0.216 on the testing set (see Figure 7).

Although LGBM performed notably better than SVR and ANN in predicting the UCS of CPB, it contains more than 100 hyperparameters, and its performance is highly sensitive to several key settings [48]. Among these, the learning rate, number of trees, and feature sampling rate are the most influential [49]. The learning rate controls the step size of gradient updates, directly affecting convergence behaviour and predictive accuracy. The number of trees governs the number of boosting iterations, balancing model performance and computational cost. The feature sampling rate improves generalisation by randomly selecting subsets of input variables, thereby mitigating overfitting. To further enhance the stability and predictive accuracy of the LGBM model, a metaheuristic optimisation algorithm was employed to fine-tune these key hyperparameters. The optimisation results are presented in the following section.

### 4.4. Result of GWO–LGBM

**Figure 8:**
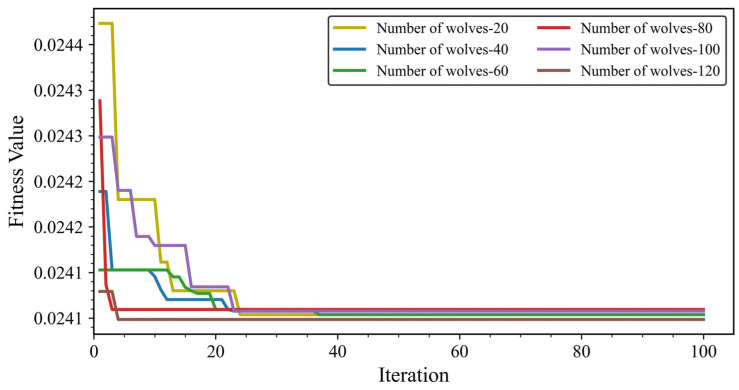
Predictive performance of the GWO–LGBM model for various populations.

During the hyperparameter search of the LGBM using the GWO algorithm (see Figure 8), the fitness curves corresponding to different population sizes stabilise after approximately 60 iterations. The optimisation results further show that the identified optimal parameters vary with population size. Specifically, when the number of grey wolves is 20, the optimal parameters are learning_rate = 0.07, n_estimators = 164, and colsample_bytree = 0.825. With a population of 40, the optimal values are learning_rate = 0.087, n_estimators = 163, and colsample_bytree = 0.82. For a population of 60, the best-performing parameters are learning_rate = 0.09, n_estimators = 164, and colsample_bytree = 0.815. When the population increases to 80, the optimal configuration becomes learning_rate = 0.087, n_estimators = 163, and colsample_bytree = 0.82. For 100 grey wolves, the parameters converge to learning_rate = 0.05, n_estimators = 172, and colsample_bytree = 0.598. Finally, with a population of 120, the optimal parameters are learning_rate = 0.084, n_estimators = 135, and colsample_bytree = 0.768.

Overall, the results indicate that a population size of 60 leads to relatively stable hyperparameter configurations and predictive performance with minimal variation. Considering both computational cost and time efficiency, a population size of 60 was therefore selected. The corresponding optimal hyperparameters—learning_rate = 0.09, n_estimators = 164, and colsample_bytree = 0.815—were adopted for the final GWO–LGBM model. Under this configuration, the model demonstrates excellent capability in predicting the UCS of CPB. Its performance on the training dataset was *R*^2^ = 0.907, *RMSE* = 0.779, *MAE* = 0.515, and *MAPE* = 0.161, while the testing dataset achieved *R*^2^ = 0.951, *RMSE* = 0.619, *MAE* = 0.371, and *MAPE* = 0.109.

**Figure 9:**
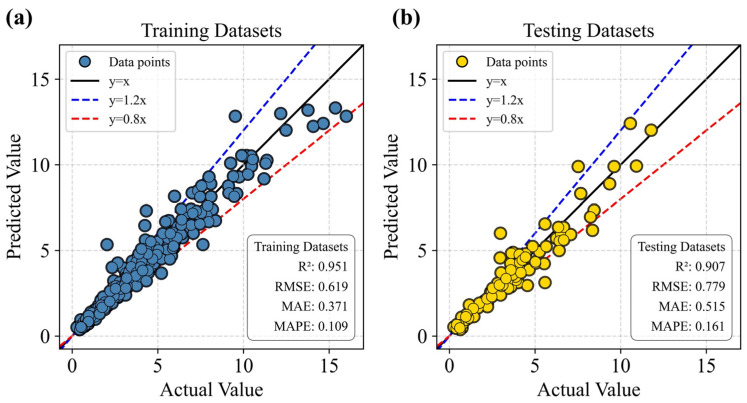
Scatter regression analysis of the GWO–LGBM model. (**a**) Training Datasets; (**b**) Testing Datasets.

To further assess the predictive reliability of the GWO–LGBM model, a dispersion analysis of its regression performance was conducted to examine the correlation between the predicted and measured values. The left panel of Figure 9 illustrates the model performance on the training dataset, whereas the right panel presents the corresponding results for the testing dataset. The solid black line denotes the ideal prediction line (y = x), and data points lying closer to this line indicate higher predictive accuracy. To provide a clearer visual representation of the error margin, two auxiliary dashed lines are added to indicate the ±20% deviation range from the ideal prediction.

It was observed that the majority of the samples (a total of 487 data) are distributed close to the ideal line and predominantly fall within the ±20% interval. This distribution pattern confirms that the GWO–LGBM model exhibits strong predictive consistency and robustness across both the training and testing datasets.

### 4.5. SHAP Feature Contribution Analysis

Although the GWO–LGBM model demonstrates excellent predictive accuracy, the internal decision-making mechanisms of such ensemble models often remain difficult to interpret in practical applications. SHapley Additive exPlanations (SHAP) was used to quantify each feature’s contribution, providing a clearer understanding of how input variables affect the model’s predictions.

SHAP is based on the Shapley value concept from cooperative game theory and provides a consistent framework for interpreting complex machine-learning models [50]. Its advantages include: (1) offering clear visual representations that highlight the features with the greatest influence on the model output; (2) enabling the interpretation of individual predictions, which helps reveal the rationale behind specific model decisions; and (3) identifying anomalous samples and problematic variables by examining SHAP values. The improved interpretability allows key factors influencing model performance to be identified more intuitively, and adjustments to these factors can subsequently enhance predictive accuracy.

The SHAP value for a feature *i* is computed by comparing the model output with and without that feature for all possible feature subsets. Formally, let *F* denote the full set of features and *S* any subset not containing feature *i*. The contribution of feature *i* is evaluated by the following difference:

(7) $$\phi_{i}=\sum_{S\subseteq F\left\{i\right\}}\frac{\left|S\right|!\left(\left|F\right|-\left|S\right|-1\right)!}{\left|F\right|}\left[f_{S\cup \left\{i\right\}}\left(x_{S\cup \left\{i\right\}}\right)-f_{S}\left(x_{S}\right)\right]$$

where *F* represents all available features and *S* denotes any subset of features that does not include feature *i*. $f_{S\cup \left\{i\right\}}$ represents the model that includes feature i, while $f_{S}$ represents the model without feature *i*. $x_{S}$ denotes the set of features in subset *S*. By comparing $f_{S\cup \left\{i\right\}}\left(x_{S\cup \left\{i\right\}}\right)-f_{S}\left(x_{S}\right)$, the contribution of feature i to the model prediction can be calculated. The Shapley value is the weighted average of all possible differences, representing the effect of each feature on the model output. This method provides a solid framework for quantifying the individual impact of features on predictions.

**Figure 10:**
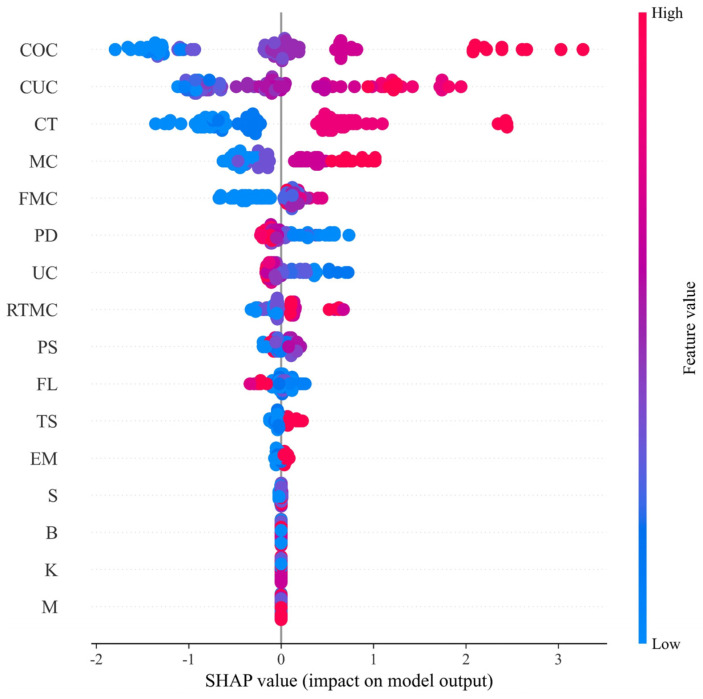
Contribution analysis of variables by SHAP beeswarm plot.

As illustrated in Figure 10, the SHAP beeswarm plot presents the influence of each feature variable on the predicted UCS of fibre-reinforced CPB. Each row corresponds to one feature, and each point represents an individual sample. The horizontal position of a point reflects its SHAP contribution value (SHAP > 0 indicates a positive contribution and SHAP < 0 indicates a negative contribution). The colour scale denotes the magnitude of the feature value (red for higher values and blue for lower values). According to the SHAP-based ranking, the features are ordered in descending importance as follows: COC, CUC, CT, MC, FMC, PD, UC, RTMC, PS, FL, TS, EM, S, B, K, and M. The results show that COC, CUC, and CT exert the most substantial influence, and higher feature values generally correspond to higher UCS. These three variables fall within the categories of “mixture proportions” and “physical properties of the tailings-cement mixture”, indicating that these two groups play dominant roles in strength development. By contrast, the contribution of fibre properties (FMC, FL, TS, EM) is relatively modest. Among these, FMC has the greatest influence, where higher FMC values enhance UCS and lower values reduce it. Among the chemical parameters, sulphur (S) is the most influential variable. Nevertheless, compared with other feature groups, chemical parameters exert only a minor effect, suggesting that it can be regarded as a low-sensitivity variable within the model.

**Figure 11:**
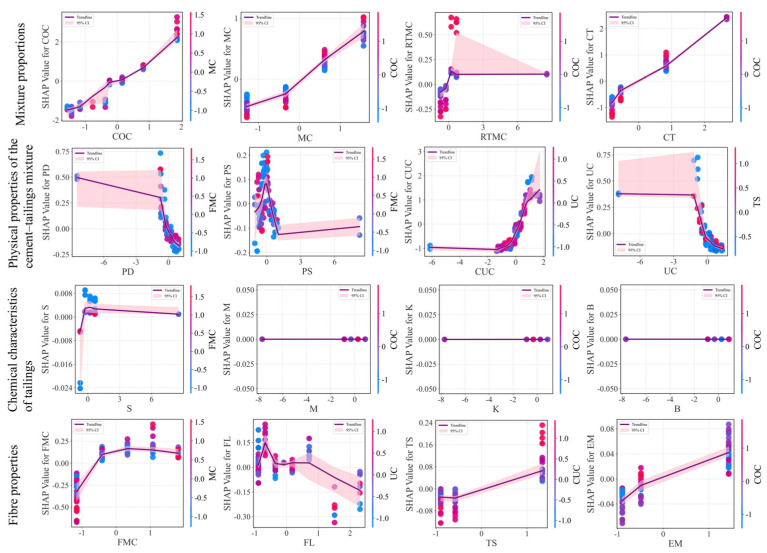
Influence analysis of variables by SHAP dependency analysis.

To further examine the relationships between the input variables and the model output, SHAP dependence plots were generated for all features (Figure 11) and interpreted according to their respective variable categories.

For the mixture proportions, COC, MC, and CT exhibit strong positive correlations with UCS, indicating that increasing cement content, mass concentration, or curing time is favourable for strength development. By contrast, RTMC demonstrates a distinct nonlinear effect. When the feature value is low (<0), its SHAP value is negative, implying a suppressive influence on UCS. As RTMC increases to approximately 0.2, its SHAP value transitions rapidly from negative to positive and reaches a peak contribution. Further increases lead to a decline and eventual stabilisation of the contribution. This pattern suggests an effective range of rod-mill tailing content: insufficient amounts fail to improve particle grading, whereas excessive amounts may disrupt the particle grading, both of which are detrimental to strength development.

For the physical properties of the cement–tailings mixture, PD and UC show the similar trend that a negative correlation with UCS, with their inhibitory effect becoming more pronounced once they exceed 0. By contrast, CUC is positively correlated with UCS, and its beneficial contribution becomes particularly notable when CUC is greater than 0.5. The influence of PS is more complex, displaying a non-monotonic relationship with UCS. Specifically, UCS reaches a local maximum when PS is close to zero, whereas both lower and higher PS values tend to suppress strength development. This observation reinforces the view that favourable particle-size distribution is one of the key factors controlling CPB strength.

For the chemical characteristics of tailings, the S displays a weak nonlinear relationship with UCS. In contrast, other chemical characteristics of tailings, such as M, K, and B, show negligible influence on UCS. The SHAP analysis indicates that a small amount of sulphur has a positive effect on UCS, whereas excessive sulphur content gradually leads to an inhibitory effect. Nevertheless, the overall magnitude is minimal (|SHAP| < 0.2), indicating that chemical properties of tailings behave largely as inert contributors within the CPB system, primarily functioning as an aggregate.

For the fibre properties, FMC shows a “strengthening-then-stabilising” pattern, suggesting a diminishing marginal benefit as its value increases. FL exhibits positive SHAP values within a moderate range (approximately −0.2 to 8), beyond which excessively long or short fibres result in reduced UCS. Both TS and EM exhibit near-linear positive correlations with UCS, indicating that increased fibre tensile strength and elastic modulus enhance the CPB.

## 5. Discussion

### 5.1. Performance Comparison

**Figure 12:**
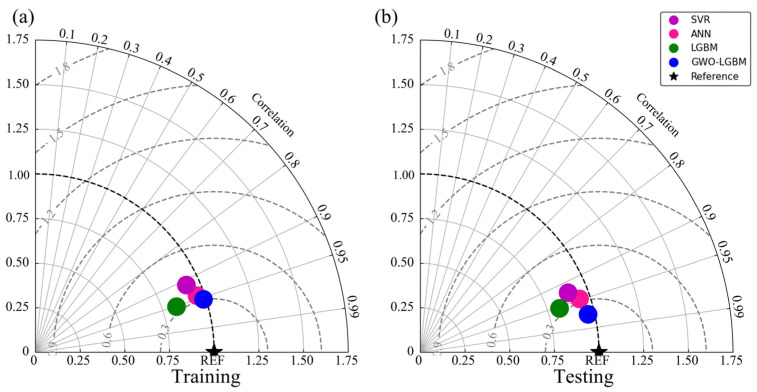
Taylor diagram comparing the performance on training and testing datasets. (**a**) Training Datasets; (**b**) Testing Datasets.

To comprehensively assess the performance of different models in predicting the UCS of CPB, a Taylor diagram was employed for comparative analysis (Figure 12). This diagram offers a unified framework for assessing model performance by displaying the standard deviation (SD), root mean square error (RMSE), and correlation coefficient (R) on the same plot. In this coordinate system, the radius denotes the variation in the model outputs, whereas the reference point (black star) corresponds to the variability in the measured data under perfect agreement (R = 1). The separation between a model marker and this reference point reflects its prediction error, with a shorter distance indicating better accuracy. Meanwhile, the angular position of each marker represents the degree of linear association with the observations, where a smaller angle implies a stronger relationship. Ideally, a model should lie as close as possible to the reference point.

In this study, the performances of four models—SVR, ANN, LGBM, and GWO–LGBM—were compared using the Taylor diagram (Figure 12). The results demonstrate that the GWO–LGBM model achieves the best performance across all three metrics: standard deviation, correlation coefficient, and RMSE, with its point located nearest to the reference, indicating strong agreement between predicted and measured UCS values. By contrast, the single machine-learning models (SVR, ANN, and LGBM) exhibit slightly lower prediction accuracy and stability. Overall, the incorporation of the GWO algorithm, which introduces an adaptive search mechanism, effectively tunes the model parameters, thereby significantly enhancing the UCS prediction performance of the LGBM model.

### 5.2. Macro and Micro Feature Verification

**Figure 13:**
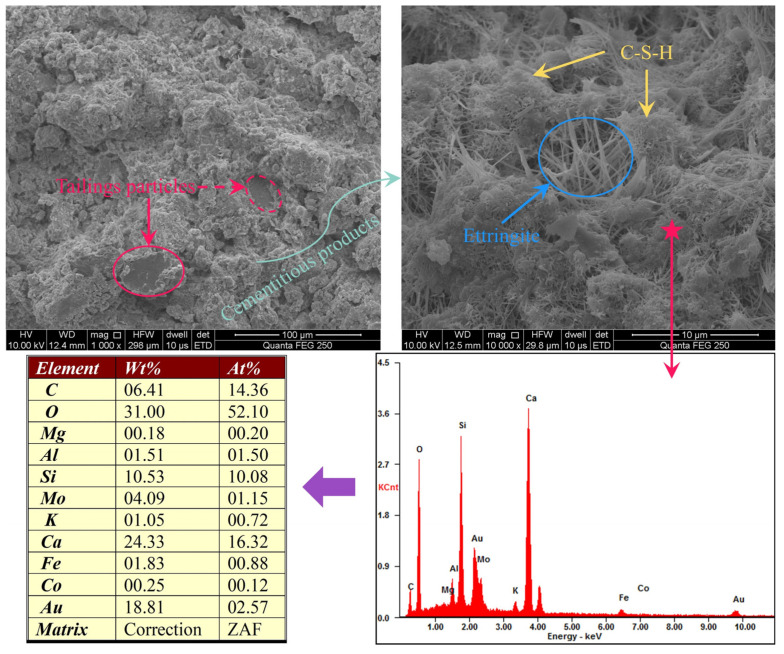
SEM microstructure of aggregates and cementitious hydration products.

Figure 13 presents the scanning electron microscopy (SEM) and energy-dispersive spectroscopy (EDS) results of the CPB specimens. It can be observed that tailings aggregates constitute the primary structural component of the CPB. Well-graded tailings help minimise voids from forming within the CPB, thereby serving as the main load-bearing structure. Moreover, hydration of the cementitious binder generates cementitious products such as hydrated gel and ettringite, which adhere to the tailings particles, enhancing structural cohesion and further increasing the CPB strength. These observations are consistent with the SHAP feature contribution analysis, which identified mixture proportions and physical properties of the cement–tailings mixture as having the most significant effects.

**Figure 14:**
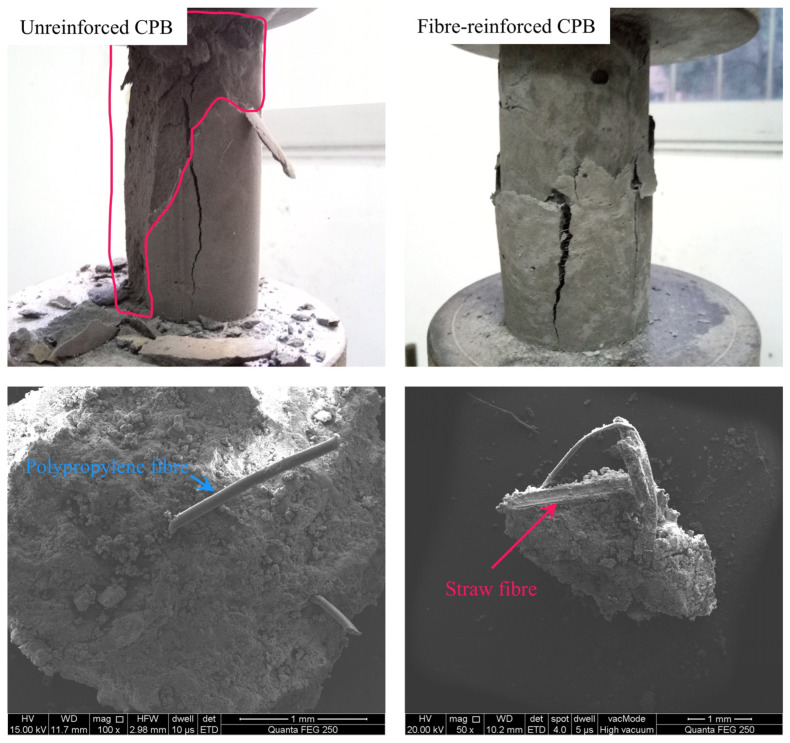
Macro- and micro-structural failure of fibre-reinforced CPB and unreinforced CPB.

Figure 14 illustrates the macro- and microstructural failure patterns of the CPB. Although SHAP feature contribution analysis suggested that fibres contribute relatively less to overall UCS, examination of specimens beyond the compressive limit reveals their beneficial effect. Unreinforced CPB exhibits extensive structural collapse with large fragments detaching, whereas fibre-reinforced CPB retains a greater degree of structural integrity after failure, demonstrating improved residual strength. It can be seen from the SEM microstructure that fibres are distributed throughout the CPB, effectively connecting dispersed particles. Their inherent tensile resistance further enhances the mechanical performance of the CPB.

### 5.3. Research Significance and Limitations

The GWO–LGBM model proposed in this study holds significant scientific and engineering value for predicting the UCS of fibre-reinforced CPB. First, compared with previous studies [26,27], the present database is expanded to include the physical and chemical properties of tailings, enabling a more comprehensive characterisation of material behaviour. Second, the model provides a high-precision and cost-effective means of predicting the UCS of CPB without abundant experiments, outperforming traditional empirical approaches and single machine-learning models in both accuracy and robustness [19,20,21,28,29]. Moreover, the model exhibits strong scalability; as the database expands, its predictive accuracy and generalisation capability are expected to improve further, allowing it to adapt to varying mine conditions, material compositions, and backfilling scenarios.

In addition, by incorporating the SHAP method, a systematic interpretability analysis is also provided in this study, revealing the contributions of different feature variables to strength development. The results indicate that cement content, curvature coefficient, and curing time play a dominant role, which is consistent with the findings reported in [13,14]. Fibre properties enhance mechanical performance by improving structural connectivity, a conclusion supported by both the experimental results of this study and previous investigations [8,9,10], whereas the chemical characteristics of tailings contribute minimally to UCS, a trend also observed in [28]. Fall et al. [15,16] reported that sulphides can promote early hydration by filling pores and enhancing strength at early ages, but may induce expansive effects at later stages, leading to strength reduction; however, such effects are highly dependent on sulphur content [17]. Moreover, while the influence of tailings’ chemical components is known to be temperature-dependent, this aspect was not addressed in the present study. Considering that the sulphur content in the CPB mixtures investigated herein was generally below 1%, the limited influence of sulphur on the UCS of CPB observed in this study is reasonable. Overall, the tailings’ chemical composition primarily acts as an inert aggregate [18]. These insights can directly inform the design and optimisation of fibre-reinforced CPB in engineering practice.

Despite these advances, several limitations remain. First, only LGBM was employed as the core learner, and a systematic comparison with other advanced models, e.g., the Gaussian process, was not conducted, so its performance advantages require further validation. Second, parameter optimisation was solely performed using GWO, without considering other metaheuristic algorithms, e.g., Particle Swarm Optimisation (PSO) or Genetic Algorithm (GA); future studies could expand on this to evaluate the applicability of different optimisation strategies. Finally, although the model input parameters are relatively comprehensive, some factors—such as different types of binders [51,52], curing temperature, and humidity [16,53,54], as well as the long-term strength of CPB—have not been considered, which may limit the model’s applicability under specific conditions.

## 6. Conclusions

After 487 UCS trials, a CPB database was established. The combination of experiments with GWO–LGBM and SHAP analyses led to the following conclusions:

1. Adding both polypropylene and straw fibre can effectively improve the UCS of CPB. The addition of fibres effectively bridged the dispersed particles of the CPB, allowing the CPB to maintain relative integrity after failure and improving its residual strength.
2. The GWO algorithm effectively optimised the key hyperparameters of the LGBM model, enhancing its stability and predictive accuracy. Compared with the SVR, ANN, and LGBM models, the GWO–LGBM model achieved a higher prediction accuracy for UCS (training set: R^2^ = 0.907, RMSE = 0.78, MAE = 0.515, MAPE = 0.157; testing set: R^2^ = 0.949, RMSE = 0.627, MAE = 0.38, MAPE = 0.115).
3. Cement content, curvature coefficient, and curing time are the key factors controlling CPB strength, and all showed positive correlations with UCS. These variables jointly shaped the overall skeleton of the CPB.
4. The alkalinity coefficient of the tailings exhibited a weak nonlinear relationship with UCS and the chemical composition of tailings acted largely as an inert contributor to the UCS of CPB, mainly providing aggregate support.

## Figures and Tables

**Table 1:** Chemical composition of the tailings [30].

Chemical Composition	Al_2_O_3_	SiO_2_	MnO	Fe_2_O_3_	MgO	CaO	K_2_O	TiO_2_	P_2_O_5_	S	Cu
Classified tailings	5.69	16.80	0.19	4.57	3.51	65.2	1.49	0.37	0.05	0.910	0.010
Rod-mill tailings	5.21	24.60	0.28	22.50	1.63	32.0	1.18	0.24	0.06	10.70	0.013
**Chemical Composition**	**Sr**	**Cr**	**Ni**	**Zn**	**Pb**	**Ba**	**Cl**	**F**	**Zr**	**As**	**Rb**
Classified tailings	0.032	0.098	0.008	0.732	0.228	/	0.018	/	0.008	/	0.006
Rod-mill tailings	0.022	0.076	0.009	0.448	0.47	0.061	0.019	0.337	0.007	0.084	0.004

## Data Availability

The original contributions presented in this study are included in the article. Further inquiries can be directed to the corresponding author.
